# Amyloid related cerebral microbleed and plasma Aβ40 are associated with cognitive decline in Parkinson’s disease

**DOI:** 10.1038/s41598-021-86617-0

**Published:** 2021-03-29

**Authors:** Hsin-Hsi Tsai, Li-Kai Tsai, Yen-Ling Lo, Chin-Hsien Lin

**Affiliations:** 1 Department of Neurology, National Taiwan University Hospital Bei-Hu Branch, Taipei, Taiwan; 2 Department of Neurology, National Taiwan University Hospital, Taipei, 100 Taiwan

**Keywords:** Biomarkers, Diseases, Neurology

## Abstract

Cerebral microbleeds (MBs) have been found in patients with cognitive decline. We aimed to examine whether MBs are associated with motor or cognitive decline in patients with Parkinson’s disease (PD). We enrolled 135 PD patients and 34 healthy controls. All participants underwent brain MRI and plasma biomarker assays, including tau, Aβ42, Aβ40, and α-synuclein. PD with dementia (PDD) was operationally defined as Mini-Mental State Examination (MMSE) score < 26 and advanced motor stage was defined as Hoehn-Yahr stage ≥ 3 during “on” status. The association between MBs and disease severity was examined using multivariate logistic regression models. More lobar MBs were observed in PD patients than controls (20.7% vs. 3.3%, *p* = 0.031). PDD patients had more lobar MBs (33.3% vs. 15.6%, *p* = 0.034), more white matter hyperintensity (*p* = 0.021) and reduced hippocampal volume (*p* = 0.001) than PD with normal cognition. The presence of lobar MB (odds ratio = 2.83 [95% confidence interval 1.04–7.70], *p* = 0.042) and severe white matter hyperintensity (3.29 [1.21–8.96], *p* = 0.020) was independently associated with PDD after adjusting for vascular risk factors and other confounders. Furthermore, plasma Aβ40 levels were associated the MMSE score (*p* = 0.004) after adjusting for age and sex. Our findings demonstrated that lobar MBs, reduced hippocampal volume, and elevated plasma Aβ40 levels are associated with PDD.

## Introduction

Parkinson’s disease (PD) is one of the most common neurodegenerative disorders, manifesting with progressive motor symptoms, such as resting tremor, bradykinesia, rigidity, and postural instability^1^. Patients with PD deteriorate not only in their motor aspects, but also in cognitive function, which have significantly impact quality of life, health-related costs, and caregiver burden^2^. Previous cross-sectional studies have shown a prevalence of dementia of nearly 30% in PD patients^3^, and people with PD have 3 to 6-times higher risk of developing dementia compared to age-matched subjects who do not have PD^4^. Identifying easily accessible markers that reflect disease progression and predict risk of PD with dementia (PDD) are needed for future mechanism-targeted therapeutic responses.

The neuropathology underlying PDD is heterogenous in nature. In addition to a more extensive cortical Lewy body pathology, several post-mortem studies in patients with PDD showed substantial proportion of patients harboring Alzheimer pathology of Aβ plaques and tau neurofibrillary tangles^5,6^. One cohort study even reported the presence of microvasculopathy lesions in 94% of patients with PDD compared to roughly 50% in PD^7^. These findings from previous literature suggest that in addition to the contribution of limbic and cortical spreading of Lewy body pathology to PDD^8^, several other mechanisms have been proposed for cognitive decline in PD. Coexisting cerebral small vessel disease (SVD) could be one of the important contributors, with previous studies showing associations between cognitive function and neuroimaging or pathological evidence of SVD in PD patients^9,10^. Cerebral microbleed (MB), which can be visualized using blood-sensitive magnetic resonance imaging (MRI) and corresponds to clusters of hemosiderin-laden macrophages on pathology, is one of the characteristic features of SVD^11^, MBs in the cortical region (lobar MBs) have been shown to be risk factors for cognitive impairment in the aging population^12^. However, the role of MBs in disease progression, especially the development of PDD, in patients with PD remains unclear. In this study, we examined whether MBs are associated with risk and severity, especially cognitive decline, in patients with PD. We also investigated the plasma levels of biomarkers related to MBs, including amyloid β42 (Aβ42) and amyloid β40 (Aβ40), to elucidate their contributions to PDD.

## Results

### Prevalence of lobar MB

**Figure 1:**
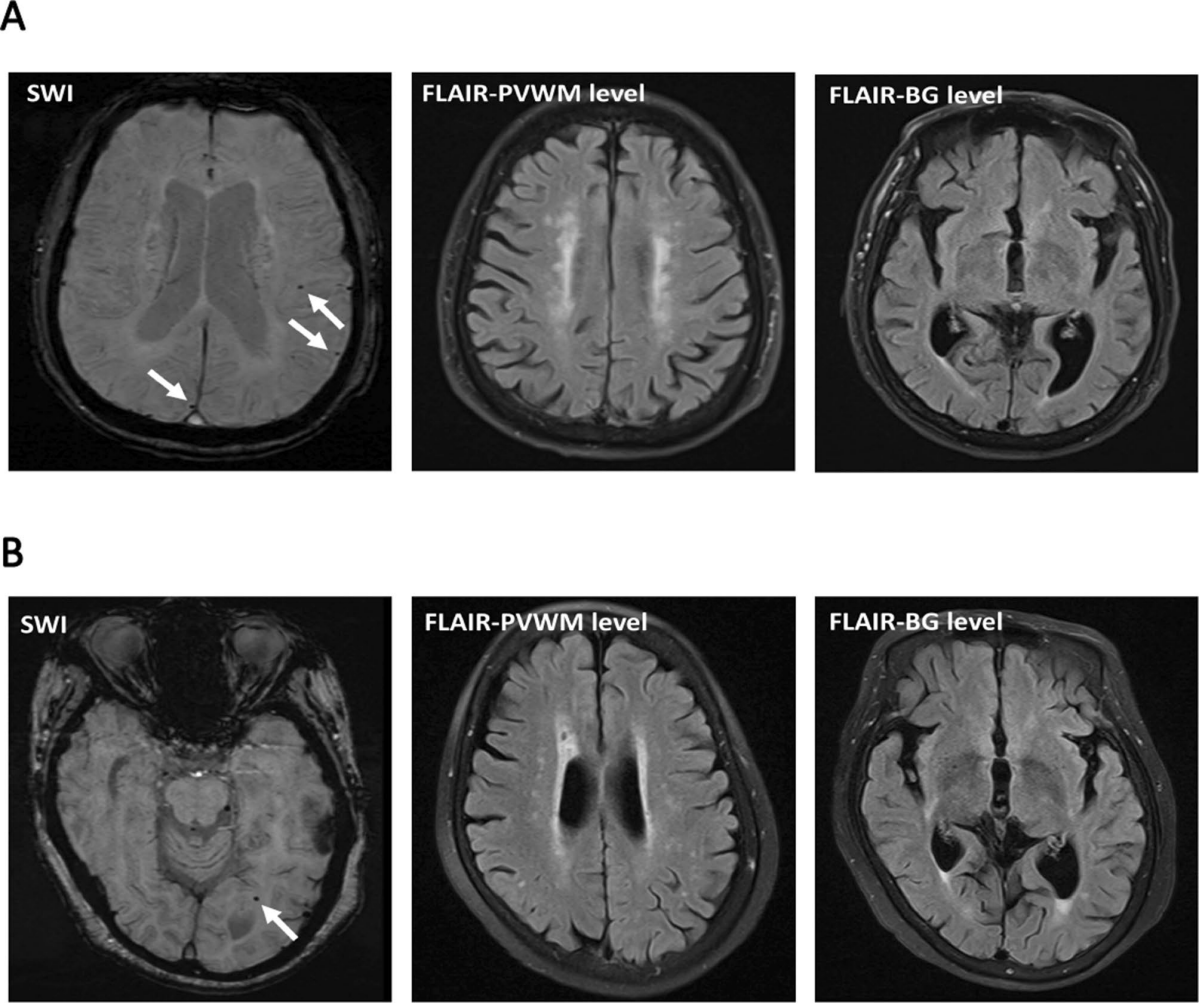
Representative images of lobar microbleeds in Parkinson’s disease. (**A**) A 66-year-old woman with Parkinson’s disease dementia (PDD, MMSE score 23) had multiple lobar microbleeds in the left parietal and right occipital lobes (arrow) and minimal white matter hyperintensity (WMH) on fluid-attenuated inversion recovery imaging. There was no lacunar infarct over bilateral BG. (**B**) An 82-year-old woman with PDD (MMSE score 16) had one lobar microbleeds in the left occipital (arrow), the left parietal, and the right temporal lobes (not shown). Mild WMH was also seen on fluid-attenuated inversion recovery imaging. There was no lacunar infarct over bilateral BG. *SWI* susceptibility weighted imaging, *FLAIR* fluid-attenuated inversion recovery imaging, *PVMW* periventricular white matter, *BG* basal ganglia.

A total of 135 PD patients (mean age 67.6 ± 11.1 years) and 34 neurologically normal controls (mean age 67.6 ± 7.9 years) were included in the analysis. We found no significant differences in vascular risk factors and baseline characteristics between PD patients and controls (Table 1). The prevalence of lobar MBs was higher in PD patients than controls (20.7% vs. 2.9%, *p* = 0.011) (Fig. 1A,B). A higher prevalence of deep MB (8.1% vs. 5.9%, *p* = 1.00) or infratentorial MB (7.4% vs. 2.9%, *p* = 0.70) was noted in PD patients than controls although the difference did not reach the statistical significance. There was no difference in the proportion of moderate to severe WMH (Fazekas scale ≧ 2) between PD and normal controls (28.1% vs 29.4%, *p* = 1.00).

**Table 1:** Comparison of microbleed in patients with PD and healthy controls.

	Control (n = 34)	PD (n = 135)	*p* value
**Basic characteristics**			
Male, N (%)	13 (38.2%)	77 (57.0%)	0.056
Age, years	67.6 ± 7.9	67.6 ± 11.1	0.995
Hypertension	17 (50.0%)	51 (37.8%)	0.241
Diabetes Mellitus	4 (11.8%)	16 (11.9%)	1.000
Dyslipidemia	7 (20.6%)	18 (13.3%)	0.288
3 T MRI scanner, N (%)	28 (82.4%)	94 (69.6%)	0.198
Disease duration (years)	N.A	9.3 ± 3.2	N.A
Hoehn-Yahr stage (on)	N.A	1.8 ± 0.7	N.A
Hoehn-Yahr stage (off)	N.A	3.2 ± 0.9	N.A
UPDRS part III (on)	N.A	21.2 ± 6.1	N.A
UPDRS part III (off)	N.A	39.5 ± 7.3	N.A
Mean LEDD (mg/day)	N.A	623.8 ± 380.1	N.A
**Presence of cerebral microbleed**	2 (5.9%)	37 (27.4%)	0.006
Presence of lobar MB	1 (2.9%)	28 (20.7%)	0.011*
Presence of deep MB	2 (5.9%)	11 (8.1%)	1.000
Presence of infratentorial MB	1 (2.9%)	10 (7.4%)	0.696
**White matter hyperintensity**			
Fazekas scale ≥ 2	10 (29.4%)	38 (28.1%)	1.000

Values are given as mean ± standard deviation or n (%).

“On” state was defined as 60–90 min after the administration of dopaminergic medications and “Off” state was defied as discontinuation of any form of dopaminergic medications for at least 12 h (usually an overnight withdrawal).

*N* number, *PD* Parkinson’s disease, *MB* microbleed, *UPDRS* Unified Parkinson's Disease Rating Scale, *LEDD* levodopa equivalent dose, *N.A*. not available.

### Comparison of PD patients with different motor and cognitive severity

Among PD patients (n = 135), the prevalence of lobar and deep MBs was comparable between patients with early-stage and advanced-stage of motor symptoms of disease (Table 2). Of 135 PD patients, 39 (28.9%) were diagnosed with PDD. A comparison of the demographics and neuroimaging characteristics between PD patients with normal cognition (MMSE 28.0 ± 1.3) and those with PDD (MMSE 21.0 ± 4.1) was shown in Table 2. Patients with PDD were older (age 72.0 ± 10.8 years vs. 65.8 ± 10.7, *p* = 0.003) and had worse motor function (UPDRS Part III 26.9 ± 11.5 vs. 19.6 ± 9.9, *p* < 0.001) than PD patients with normal cognition. In neuroimaging analysis, patients with PDD had higher prevalence of lobar MBs (33.3% vs. 15.6%, p = 0.034), more severe WMH (7.0 ± 7.7 vs. 3.9 ± 5.3 mL, *p* = 0.021), reduced hippocampal volume (3437 ± 492 vs. 3757 ± 496 mm^3^, *p* = 0.001), and thinner cortical thickness in the frontal, temporal, and parietal lobes (all *p* < 0.05) than PD patients with normal cognition.

**Table 2:** Demographics and image characteristics in Parkinson’s disease patients.

	Early motor stage of PD (n = 121)	Advanced motor stage of PD (n = 14)	*p* value	PD with normal cognition (n = 96)	PD with dementia (n = 39)	*p* value
Male	67 (55.4%)	10 (71.4%)	0.393	53 (55.2%)	24 (61.5%)	0.57
Age, years	64.8 ± 14.9	68.6 ± 10.6	0.120	65.8 ± 10.7	72.0 ± 10.8	< 0.01**
Hypertension	45 (37.2%)	6 (42.9%)	0.773	33 (34.4%)	18 (46.2%)	0.24
Diabetes	14 (11.6%)	2 (14.3%)	0.672	11 (11.5%)	5 (12.8%)	0.78
Dyslipidemia	17 (14.0%)	1 (7.1%)	0.692	14 (14.6%)	4 (10.3%)	0.59
MMSE	26.5 ± 3.6	21.5 ± 6.1	< 0.01**	28.0 ± 1.3	21.0 ± 4.1	< 0.01**
UPDRS Part III motor score (on)	19.6 ± 9.0	40.8 ± 6.3	< 0.001**	19.6 ± 9.9	26.9 ± 11.5	< 0.01**
LEDD (mg/day)	571.5 ± 346.7	1099.4 ± 317.2	< 0.001	586.1 ± 383.5	724.7 ± 363.5	0.054
3 T MRI scanner, N (%)	84 (69.4%)	10 (71.4%)	1.000	67 (69.8%)	27 (69.2%)	1.000
**Cerebral microbleed**	33 (27.3%)	4 (28.6%)	1.000	22 (22.9%)	15 (38.5%)	0.09
Lobar MB	27 (22.3%)	1 (7.1%)	0.299	15 (15.6%)	13 (33.3%)	0.03*
Deep MB	9 (7.4%)	2 (14.3%)	0.319	5 (5.2%)	6 (15.4%)	0.08
Infratentorial MB	9 (7.4%)	1 (7.1%)	1.000	7 (7.3%)	3 (7.7%)	1.000
**White matter hyperintensity**						
Fazekas scale ≥ 2	35 (28.9%)	3 (21.4%)	0.757	20 (20.8%)	18 (46.2%)	< 0.01**
Median volume, mL (± IQR)	4.8 ± 6.1	4.6 ± 7.1	0.923	3.9 ± 5.3	7.0 ± 7.7	0.02*
Caudate volume, mm^3^	3207 ± 563	2915 ± 476	0.065	3193 ± 547	3134 ± 594	0.58
Hippocampus volume, mm^3^	3703 ± 494	3343 ± 579	0.013*	3757 ± 496	3437 ± 492	< 0.01**
**Cortical thickness, mm**	2.34 ± 0.28	2.27 ± 0.12	0.382	2.36 ± 0.29	2.27 ± 0.18	0.10
Frontal cortical thickness	2.39 ± 0.21	2.29 ± 0.17	0.085	2.41 ± 0.20	2.31 ± 0.21	0.01*
Temporal cortical thickness	2.75 ± 0.24	2.64 ± 0.21	0.126	2.77 ± 0.23	2.64 ± 0.24	< 0.01**
Parietal cortical thickness	2.16 ± 0.18	2.07 ± 0.10	0.080	2.17 ± 0.17	2.09 ± 0.18	0.01*
Occipital cortical thickness	1.81 ± 0.19	1.76 ± 0.09	0.265	1.82 ± 0.18	1.77 ± 0.17	0.11

Values are given as mean ± standard deviation or n (%) unless otherwise noted. Hoehn-Yahr stage < 3 was defined as early motor stage of PD, and stage ≥ 3 was defined as advanced motor stage of PD.

*N* number, *IQR* interquartile range, *MB* microbleed, *PD* Parkinson’s disease, *MMSE* mental state examination, *UPDRS* Unified Parkinson's Disease Rating Scale, *LEDD* levodopa equivalent dose.

*p < 0.05; **p < 0.01.

Table 3 showed results from multivariable regression models investigating the neuroimaging markers in predicting PDD. Only the presence of lobar MBs (odds ratio [OR] 5.19 [95% confidence interval 1.49–8.12], *p* = 0.010) was independently associated with PDD after adjusting for age, sex, vascular risk factors (hypertension, diabetes, hyperlipidemia), H-Y stage, LEDD, WMH volume and MRI scanner filed strength (1.5 T or 3 T).

**Table 3:** Multivariable models for cerebral small vessel disease markers in predicting PD dementia.

PD cognitive impairment	OR (95% CI)	*p* value
Presence of any MB	2.13 (0.72–6.29)	0.172
Presence of lobar MB	5.19 (1.49–8.12)	0.010
Presence of deep MB	0.91 (0.18–4.72)	0.910
Presence of infratentorial MB	0.53 (0.09–3.05)	0.474

*Adjusted for age, sex, vascular risk factors, Hoehn-Yahr stage, levodopa equivalent dose, white matter hyperintensity volume, and MRI scanner field strength (1.5 T/3 T).

### Plasma biomarkers in PD patients with different motor and cognitive severity

**Figure 2:**
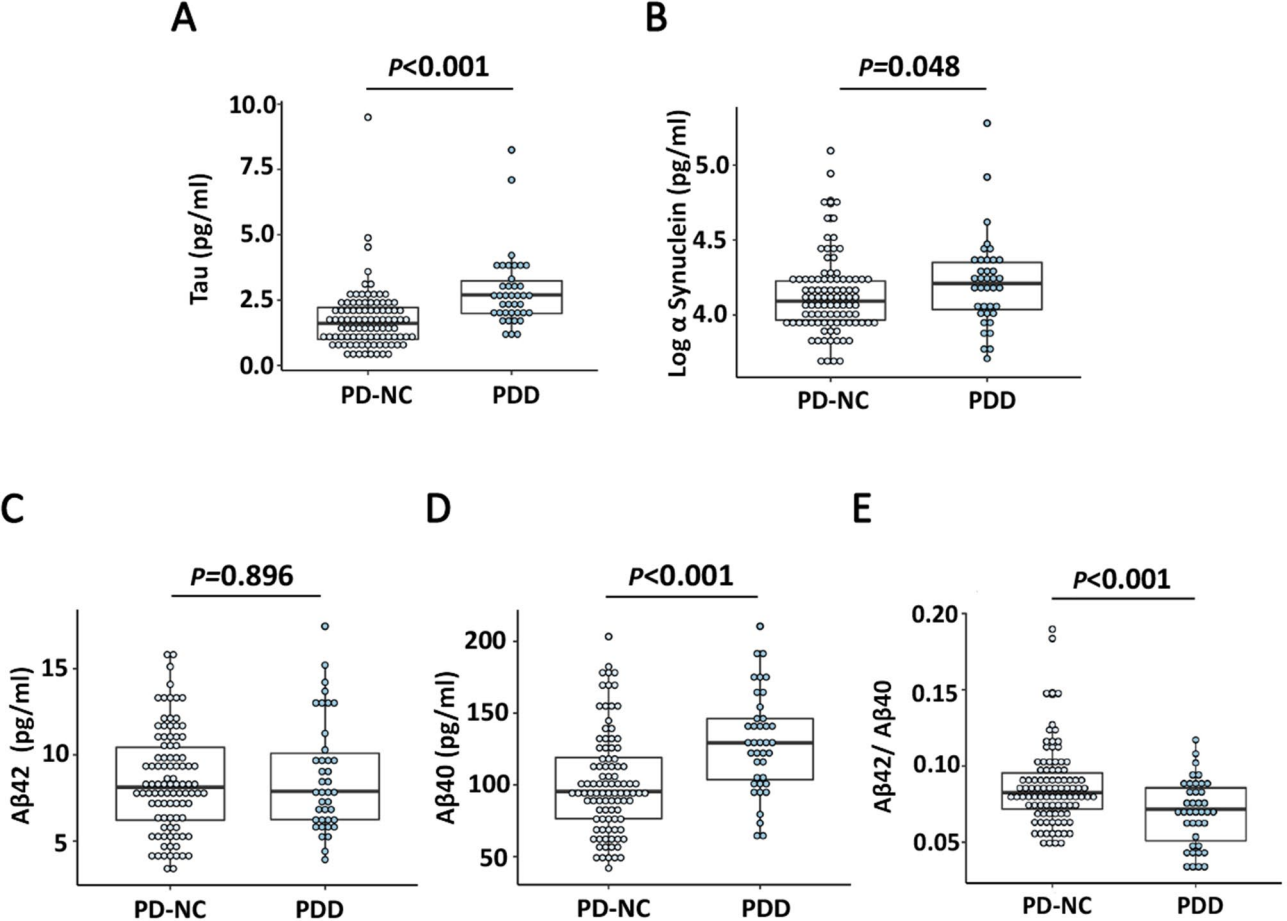
Comparison of plasma biomarkers between Parkinson’s disease patients with normal cognition and dementia. Plasma levels of (**A**) total tau, (**B**) α-Synuclein (log transformed), (**C**) Aβ42, (**D**) Aβ40, and (**E**) Aβ42/Aβ40 ratio in PD patients with normal cognition and with dementia. *PD-NC* Parkinson’s disease with normal cognition, *PDD* Parkinson’s disease dementia. *p < 0.05; **p < 0.01.

Figure 2 shows the plasma levels of total tau, Aβ40, Aβ42, and α-synuclein in PD patients with different disease severity. Compared to PD patients with normal cognition, patients with PDD had similar plasma Aβ42 levels (median 7.89 [IQR 6.22–10.29] vs. 8.13 [IQR 6.19–10.57] pg/mL, *p* = 0.896), but higher levels of total tau (2.70 [IQR 1.94–3.31] vs. 1.61 [IQR 0.99–2.24] pg/mL, p < 0.001), Aβ40 (129.23 [IQR 103.24–147.70] vs. 95.37 [IQR 75.91–119.43] pg/mL, *p* < 0.001), and α-synuclein (16,247 [IQR 10,802–22,546] vs. 12,385 [IQR 9244–16852] pg/mL, *p* = 0.048). In addition, the ratio of Aβ42 to Aβ40 (Aβ42/40) was lower in PDD than PD with normal cognition (0.07 ± 0.02 vs. 0.09 ± 0.03, *p* < 0.001). The association between PDD and plasma levels of total tau, Aβ40, and the Aβ42/40 ratio remained significant after adjusting for age and sex (all *p* < 0.05).

We further investigated the associations between the abovementioned plasma markers and clinical or radiological biomarkers (Table 4). We found that MMSE score was independently associated with plasma levels of total tau (standardized β = − 0.33, *p* < 0.001) and Aβ40 (standardized β = − 0.25, *p* = 0.004), and the Aβ42/40 ratio (β = 0.25, *p* = 0.006) after adjusting for age and sex. In addition, the plasma level of Aβ40 was independently associated with hippocampal volume (β = − 0.25, *p* = 0.006) after adjusting for age and sex.

**Table 4:** The associations between plasma biomarkers and clinical or neuroimaging characteristics.

	a-Synuclein		total Tau		Aβ40		Aβ42/Aβ40	
	Standardized β	*p* value	Standardized β	*p* value	Standardized β	*p* value	Standardized β	*p* value
MMSE score	0.02	0.875	− 0.33	< 0.001**	− 0.25	0.004**	0.25	0.006**
UPDRS motor score (on)	0.02	0.788	0.11	0.241	0.15	0.078	− 0.16	0.079
Hippocampal volume	0.04	0.664	− 0.12	0.246	− 0.25	0.006**	0.07	0.484
Caudate volume	− 0.04	0.662	− 0.07	0.455	− 0.17	0.061	0.12	0.217
Global cortical thickness	0.09	0.361	0.01	0.943	− 0.11	0.224	0.12	0.214
Frontal thickness	0.10	0.283	0.08	0.422	− 0.09	0.350	− 0.06	0.517
Lobar MB	0.02	0.869	0.12	0.194	− 0.03	0.729	− 0.04	0.619
Deep MB	− 0.09	0.293	0.04	0.687	0.03	0.717	− 0.06	0.508
WMH volume	− 0.08	0.379	0.05	0.589	− 0.01	0.936	− 0.05	0.597

Analysis was adjusted for age and sex.

*MB* microbleed, *MMSE* mini-mental state examination, *UPDRS* Unified Parkinson's Disease Rating Scale, *WMH* white matter hyperintensity.

## Discussion

Our results demonstrated a higher prevalence of lobar MBs in PD compared to healthy controls. In PD patients, more lobar MB was observed in patients with PDD than PD with normal cognition, but the number of lobar MBs was similar between patients with different motor severity. Furthermore, the presence of lobar MBs was independently associated with PDD. The cognitive severity, defined by MMSE score and hippocampal volume, was associated with increased plasma Aβ40. Our findings suggest that, in addition to plasma biomarkers, lobar MBs may serve as a surrogate radiological marker for cognitive decline in PD.

MBs are small, round, hypointense lesions visible on T2*-weighted gradient-recalled echo or SWI sequences^11^. Aging and cerebral SVD caused by hypertension or cerebral amyloid angiopathy are the most common risk factors for cerebral MBs^11,13^. The prevalence of MBs in PD patients has been reported to range from 15 to 20%^14–17^, which is similar to the prevalence in our study (20.7%). Recent studies with fewer patients have consistently shown that MBs may be associated with cognitive decline in PD^17,18^. Our results were not only in line with previous findings, but extended the knowledge that MBs, particularly in lobar regions, are more frequently found in PD patients than controls. The presence of lobar MBs, but not deep or infratentorial MBs, was independently associated with risk of PDD, suggesting that microvascular pathology in the cortical vessels may contribute to the cognitive dysfunction in PD patients. Limited evidence is available on the pathophysiology between lobar MBs and cognitive impairment in PD. A few post-mortem studies have revealed evidence of increased amyloid angiopathy in the leptomeningeal vessels in PD patients compared to controls^19–21^. Our SWI imaging results further support these pathology findings and raise the hypothesis that microvascular injury caused by amyloid deposition within cortical vascular walls could contribute, at least in part, to the development of PDD.

In addition to lobar MBs, we also demonstrated a strong association between WMHs and cognitive impairment in PD patients. WMHs generally reflect white matter tissue ischemia, and are regarded as an imaging marker for vascular risk factors^22^. Previous studies have already established WMH as a risk factor for PD cognitive decline and cerebral atrophy^23^, and highlighted the importance of modifying vascular risk factors to delay cognitive decline in PD^10,24^. Our findings of increased MBs and WMHs in patients with PDD compared to controls after adjusting vascular risk factors reinforce the importance of amyloid deposition in intracranial small vessels and the resulting microvascular injury in the pathogenesis of PDD. However, our current clinical study design could not provide pathogenic mechanisms for explaining the causality between amyloid deposition, microvascular injury and the development of cognitive decline in PD. Further studies combining advanced neuroimaging modality, including amyloid and tau positron emission tomography (PET) scans and diffusion tractography, in larger PD cohorts with longitudinal follow-ups combing post-mortem pathology studies are warranted to elucidate the deposition and trajectory of amyloid in the development of PDD.

In addition to imaging markers, our study also concomitantly assayed plasma Aβ42 and Aβ40 levels in PD patients. We found increased Aβ40, but not Aβ42, in PDD compared to PD with normal cognition. In line with our findings, a recent study found that the plasma level of Aβ40 is altered in patients with PDD compared to PD with normal cognition^25^. Coexisting Alzheimer’s disease pathology has been found in patients with PDD^26^ and could partially explain our findings of elevated plasma levels of total tau and reduced Aβ42/Aβ40 ratio in PDD. Although studies regarding plasma biomarkers in PDD are inconsistent, a higher total tau level in plasma and lower Aβ42/Aβ40 ratio are mostly associated with cerebral β amyloid deposition^25,27^. In addition to contributing to amyloid deposition, Aβ40 have been linked to white matter lesions in non-demented elderly, patients with Alzheimer’s disease, and patients with cerebral amyloid angiopathy^29,30^. In the transgenic amyloid precursor protein mouse model of Alzheimer’s disease, Aβ40, but not Aβ42, reproduced the cerebrovascular alterations and cognitive decline, pointing to a possible relationship between Aβ40 and microvascular injury in cognitive dysfunction^31^. Even though our study did not demonstrate a direct association between plasma Aβ40 levels and the presence of lobar MBs or other radiological markers of SVD, the correlation between plasma Aβ40 levels and cognitive function in PD suggests a potential contribution of cortical microvascular injury, in addition to amyloid deposition, to the cognitive decline in PD. Further functional studies in animal models or post-mortem brain pathology studies are needed to unravel the role of Aβ40 in the pathophysiology of PDD.

The current study has several limitations. First, although we applied the most commonly used clinical diagnostic criteria of PD based on the United Kingdom PD Society Brain Bank diagnostic criteria and also applied brain MRI, Tc-99 m TRODAT-1 SPECT scan and the UDPSR part III scores for measuring the participants’ responses to levodopa treatment for ensuring the diagnostic accuracy, we only excluded patients with vascular parkinsonism having basal ganglia lacunes on MRI but not those with white matter hyperintensities. This raised the limitation of this study that vascular Parkinsonism was not completely excluded and that only those with basal ganglia lacuna were excluded. Second, the cross-sectional design in this study restricts the interpretation when confirming causality between lobar MBs and cognitive impairment in PD. Whether the presence of lobar MBs could predict cognitive deterioration in PD should be validated in longitudinal follow-up studies with longer follow-up. In around 30% of our PD patients, the evaluation of MB was based on lower MRI filed strength with 1.5 T scanner, which may have lowered our sensitivity in detecting MB presence^32^. Third, cognition function was evaluated using the MMSE, a simple test of global cognitive impairment. Detailed neuropsychological tests evaluating individual cognitive domains may provide more information for assessing the impact of lobar MBs on cognitive impairment in PD. Furthermore, the relatively small sample size limits our power to determine the effect of MB topography on cognitive impairment in PD and regional cerebral atrophy, which could be of great value and explain the mechanism underlying hemorrhagic microangiopathy in PD cognitive impairment. Lastly, coexisting Alzheimer’s disease pathology or microvasculopathy could not be completely excluded in our patients with cognitive impairment, especially in those of older age. We used plasma instead of cerebrospinal fluid for the biomarker analysis, and the role of biomarkers in predicting cerebral pathology is still inconsistent. Our earlier report investigating plasma multiplex markers, including Aβ42, Aβ40 and α-synuclein, have showed that there is a significantly higher plasma α-synuclein level in patients with PD compared with age-matched controls^33^. Future studies incorporating multi-modality markers, including the in vivo molecular imaging, such as amyloid and tau PET scans, and biofluid markers are warranted to clarify the nature of the underlying pathology in these patients.

In summary, our findings revealed a higher prevalence of lobar MBs in PD patients compared to healthy controls. The presence of lobar MBs is an independent predictor of PDD and may help clinicians identify patients at risk of cognitive decline in clinical practice. The association between elevated plasma levels of Aβ40 and cognitive impairment in PD further supports amyloid-related microvascular injury as a potential contributor to the pathophysiology of PDD.

## Methods

### Study participants

This study included 169 participants, including 135 patients with PD of variable severity and 34 healthy age and sex-matched controls. All participants were recruited from the National Taiwan University Hospital (NTUH), a tertiary referral center in Taiwan. PD was diagnosed based on the United Kingdom PD Society Brain Bank clinical diagnostic criteria^34^. Patients who had symptoms and signs suggestive of atypical or secondary Parkinsonism (vascular parkinsonism, multiple system atrophy, progressive supranuclear palsy, corticobasal ganglionic degeneration, and neuroleptic agent-related parkinsonism) were excluded. In order to exclude patients with vascular parkinsonism, patients were excluded if the parkinsonism features occurred after stroke episodes or the neurological examinations showed magnetic gait pattern combined with prominent postural instability and lacking responsiveness to levodopa treatment, combining upper motor neuron signs, and there were multiple lacunes over bilateral basal ganglia on brain MRI scans. We applied the updated recommendations for clinically diagnosis of vascular parkinsonism from an expert working group^35^ to strangely exclude patients with parkinsonism in this study. In addition to brain MRI, other neuroimaging studies were performed for assisting the clinical diagnosis in selected patients, including the Tc-99 m TRODAT-1 dopamine transporter single photon emission tomography (SPECT) scan. To ensure the diagnostic accuracy, all participants underwent clinical follow-up in our movement disorder clinics for at least 3 years. Healthy controls who were cognitively normal and without neurological diseases were recruited from the same institution. Participants with previous history of strokes or cardiovascular events were not enrolled in the study. Vascular risk factors, including diabetes mellitus, hypertension, and hyperlipidemia were recorded.

Among the PD patients, motor symptom severity was evaluated using the part III motor subscale of the Unified Parkinson’s Disease Rating Scale (UPDRS Part III score)^36^ and Hoehn-Yahr (H-Y) staging^37^. The anti-parkinsonism medications using an estimate of the levodopa equivalent dose (LEDD) was recorded. Patients were excluded if the UPDSR part III score did not improve more than 30% from “OFF” state after dopaminergic medication administration. Cognition was examined using the Mini Mental State Examination (MMSE)^38^. Motor severity at H-Y stage < 3 was defined as early-stage PD, and severity at stage ≥ 3 was defined as advanced-stage PD during “on” state. PDD was diagnosed according to the recommended diagnostic criteria from the Movement Disorder Society task force^39^. PDD was operationally defined as MMSE score < 26 for identifying significant cognitive impairment in patients with PD and impairments in instrumental activities of daily living (e.g., inability to manage finances or cope in social situations). We applied MMSE score < 26 as the cut-off as a possible PDD diagnostic feature with sensitivity and specificity rates of 0.92 and 0.42, respectively^40^. This study was performed with the approval and in accordance with the guidelines of the institutional review board in NTUH (201605001MINA). All participants provided signed written informed consent before enrollment.

### Brain MRI analysis

All participants underwent structural MRI at 1.5 T or 3 T field strength (Siemens MAGNETOM Aera, Verio, TIM, or mMR; Siemens Medical Solutions, Malvern, PA). Susceptibility weighted imaging (SWI) acquisition was performed with a T2*-weighted gradient echo sequence with flip angle 15°, repetition time (TR)/echo time (TE) = 27–28/20 ms, field of view = 23–24 cm, slice thickness = 1.6–3 mm. SWI, and minimum intensity projection images were acquired by in-line post-processing of magnitude and phase images. The other protocol included T1-weighted magnetization‐prepared rapid gradient‐echo (flip angle 9–12°,TR/TE = 1630–2000/2.44–4.65 ms, field of view = 25.6 cm, slice thickness = 1 mm), T2-weighted imaging (TR/TE = 3500–3530/83–103 ms, field of view = 22–23 cm, slice thickness = 5 mm), fluid-attenuated inversion recovery imaging (TR/TE = 10,000/89–90 ms, field of view = 22–23 cm, slice thickness = 5 mm), diffusion-weighted imaging and apparent diffusion coefficient maps.

The location and number of MBs were evaluated on SWI based on previous guidelines^41^. MBs were defined as lesions with homogeneous round signal loss (< 10 mm in diameter) on SWI, but not as symmetric hypointensities and flow voids from blood vessels (Fig. 1A,B). MBs were counted in the lobar region (i.e., the frontal, temporal, parietal, occipital, and insular cortices), deep region (i.e., basal ganglia, thalamus, internal capsule, external capsule, corpus callosum, and deep periventricular white matter), and infratentorial region (i.e., brainstem and cerebellum), respectively. In addition to MBs, white matter hyperintensities (WMHs) were scored on fluid-attenuated inversion recovery images using the Fazekas scale^42^. WMH volume was also calculated using an in-house semi-automated algorithm as described previously^43^. WMH estimated by Fazekas visual rating scale has been shown near equivalent to quantitative estimates and was used extensively in neuroimaging research^44,45^. Our group previously also applied this visual rating scale in several studies investigating cerebral small vessel disease^46,47^. All MRI scans were also processed by FreeSurfer software v6.0.0 (http://surfer.nmr.mgh.harvard.edu/) for the cerebral cortex thickness, hippocampus volume, and caudate nucleus volume. Cortical surface volumetric analysis was calculated using T1‐weighted MPRAGE sequence while white matter lesions were best visualized using T2 FLAIR sequence.

### Plasma biomarker assays

Venous blood (10 mL) was drawn from each study participant at enrollment. Blood samples were centrifuged (2500*g* for 15 min) within 3 h of collection and the plasma aliquoted into cryotubes (Thermo Fisher Scientific, MA, USA) and stored at − 80 °C before testing. Plasma levels of total tau, Aβ42, Aβ40, and α-synuclein were analyzed using the Simoa immunoassay platform (Quanterix, Lexington, MA, USA) as described previously^48^. Measurements were performed by board-certified laboratory technicians who were blinded to the clinical groups. All samples were run in duplicate, and the average concentrations calculated. In addition, two internal quality control samples were run in the beginning and end of each run. The quality controls for all four biomarkers passed.

### Statistical analysis

Discrete variables were presented as proportions (%) and continuous variables as mean ± standard deviation (SD) or median (interquartile range [IQR]) as appropriate based on their distribution. Categorical variables were analyzed using Fisher’s exact test, and continuous variables using independent-samples t test (for normal distributions) or Mann–Whitney U test (for non-normal distributions). Demographics, vascular risk factors, and the prevalence of MBs were compared between PD patients and normal controls. In the PD group, clinical and radiological characteristics, and plasma levels of individual biomarkers were compared in patients with early-stage vs. advanced-stage PD, and in patients with PD with normal cognition vs. PDD. The association between MBs and PDD was further evaluated in multivariable logistic regression models with covariates including age, sex, vascular risk factors (presence/absence of hypertension, diabetes, hyperlipidemia), H-Y stage, LEDD, WMH volume and the MRI scanner filed strength (1.5 T or 3 T). For plasma biomarker analysis, plasma levels of total tau, Aβ42, Aβ40, Aβ42/40 ratio and α-synuclein were compared using both univariable and multivariable analysis for adjusting age and sex. The association between plasma biomarkers and clinical or radiological characteristics were further investigated using multivariable linear regression analyses with age and sex as covariates. All statistical analyses were performed using SPSS version 25 (IBM Corp., Armonk, NY). All tests of significance were two-tailed with a threshold for significance of p < 0.05.

### Ethics approval

The institutional ethics board committees of National Taiwan University Hospital.

### Consent for publication

All authors agree the final version of the manuscript and agree for publication.

## Data Availability

All data was available under reasonable request.
